# Comparisons of One to Three Monthly Injections of Aflibercept for Diabetic Macular Edema by Practical Protocol

**DOI:** 10.1155/2021/1374891

**Published:** 2021-02-12

**Authors:** Yuko Hayashi, Tomoaki Tatsumi, Toshiyuki Oshitari, Tomomi Kaiho, Yoko Takatsuna, Miyuki Arai, Takayuki Baba, Shuichi Yamamoto

**Affiliations:** ^1^Department of Ophthalmology and Visual Science, Chiba University Graduate School of Medicine, Chiba, Japan; ^2^Department of Ophthalmology, International University of Health and Welfare, Narita, Japan

## Abstract

The purpose of this study was to compare the efficacies of one initial intravitreal injection of aflibercept followed by a *pro re nata* (PRN; 1+PRN) regimen to those of three consecutive monthly injections followed by the PRN (3+PRN) regimen for diabetic macular edema (DME) with practical protocols. The medical records of 95 eyes of 71 cases that were diagnosed with DME and had received intravitreal aflibercept (IVA) injections were reviewed. Fifty-seven eyes had received IVA with the 1+PRN regimen, and 38 eyes had received IVA with the 3+PRN regimen. The best-corrected visual acuity (BCVA) and the central macular thickness (CMT) were measured at the baseline and at 1, 3, 6, and 12 months after the IVA. The mean number of injections of the 1+PRN group was 2.9 ± 1.7, which was significantly fewer than that of the 3+PRN group at 4.6 ± 1.4 (*P* < 0.001). The change of the mean BCVA before and after the IVA at 12 months of the 3+PRN group was −0.14 ± 0.17 logMAR units which was significantly better than that of the 1+PRN group of −0.045 ± 0.25 logMAR units (*P* = 0.02). The change of the CMT before and after the IVA at 6 months of the 3+PRN group was −141.3 ± 152.4 *μ*m which was significantly more than that of the 1+PRN group at −86.1 ± 117.8 *μ*m (*P* = 0.013). Although the mean number of injections was more than that in the 1+PRN regimen, the 3+PRN regimen had better visual outcomes at 12 months. In a practical protocol, we recommend the 3+PRN regimen for patients with DME (IRB#3541).

## 1. Introduction

Diabetic macular edema (DME) is the most common cause of vision decrease in patients with nonproliferative diabetic retinopathy [1]. An earlier meta-analysis of 22,896 diabetic patients found that the prevalence of DME was 6.81% of patients with diabetes mellitus [2]. Although there are several therapeutic procedures to treat DME including focal/grid laser photocoagulation, intravitreal or sub-Tenon triamcinolone acetonide (TA) injections [3], pars plana vitrectomy [4], and intravitreal dexamethasone implant [5–9], intravitreal injections of antivascular endothelial growth factor (anti-VEGF) agents have become the gold standard therapy for DME worldwide [10–17]. Evidence from the results of several clinical trials suggested that repeated intravitreal anti-VEGF agent injections significantly improved the visual acuities and maintained the visual outcomes for a long period in eyes with DME [10–17]. However, the medical cost has severely restrained the frequent injections of anti-VEGF agents for most patients [18]. In Japan, the cost of a single intravitreal injection of aflibercept is approximately $1,500, and most patients have to pay 30% of the medical costs at every visit to the hospital in addition to the annual medical insurance fees. Thus, a reduction in the number of injections would be a practical improvement.

The results of our recent studies of the different injection protocols indicated that as few as 3.8 ± 2.4 anti-VEGF injections can significantly improve the best-corrected visual acuity (BCVA) and the central macular thickness (CMT) in eyes with DME after a one-year treatment period [19]. In representative clinical trials including the VISTA and the VIVID studies, the mean number of injections was 9-12 times/year. Thus, the lower number of aflibercept injections was more cost effective in resolving the DME in real-world studies. In addition, our previous real-world study showed that the effects of the fewer number of intravitreal aflibercept (IVA) injections persisted longer than those of intravitreal ranibizumab (IVR) injections [20]. Thus, we have recently selected IVA for the treatment of DME with a practical protocol in the Chiba University Hospital.

In these studies, the injection protocol was 1 to 3 consecutive monthly injections, but if the CMT was >300 *μ*m, additional injections were given [19, 20]. A reduction of the total number of IVA injections is more desirable for the treatment of DME with a practical protocol because it improved the cost effectiveness. We have recently compared the effectiveness of one initial injection of anti-VEGF agents followed by the *pro re nata* (PRN; 1+PRN) regimen to that of three monthly injections followed by the PRN (3+PRN) regimen for macular edema (ME) associated with a branch retinal vein occlusion (BRVO) [21]. The results of that study indicated that the improvements of the BCVAs and the CMTs were not significantly different between the 1+PRN and the 3+PRN groups [21]. However, the total number of injections after 12 months in the 1+PRN group was 3.1 ± 1.6 times/year which was significantly fewer than that in the 3+PRN group at 5.1 ± 1.7 times/year [21]. Thus, we recommended the 1+PRN protocol for patients with ME associated with BRVO instead of the 3+PRN protocol [21].

Ebneter et al. compared two treatment regimens with IVR for DME: one with the treat-and-extend regimen (TER) and the other with the PRN regimen [22]. Their results indicated that the visual outcomes were similar, but the number of injections was fewer with the PRN regimen than with the TER [22]. Thus, the PRN protocol can be more cost effective in the real world. James et al. compared the visual outcomes after IVR for DME in the 1+, 2+, and 3+PRN regimens with a practical protocol [23]. They concluded that one-third of the eyes with DME significantly improved with the 1+PRN regimen and the 2+PRN regimen [23]. Thus, they suggest that if baseline visual acuity is good, the 3+PRN regimen should not be used [23]. In a PubMed search, we did not extract any real-world study comparing efficacies of IVA for DME with the 1+PRN to the 3+PRN regimen.

Thus, the purpose of this study was to compare the effectiveness of treating eyes with DME by the 1+PRN regimen to that by the 3+PRN regimen on the BCVA and CRT.

## 2. Patients and Methods

The medical records of 95 eyes of 71 cases that were diagnosed with DME and had received the first IVA injection at the Chiba University Hospital from January in 2015 to December in 2017 were reviewed. The inclusion criteria were patients with DME who had a vision impairment and a CMT > 250 *μ*m based on the optical coherence tomographic images (SD-OCT, Heidelberg Engineering, Heidelberg, Germany) [19, 20, 24]. The recent Japanese survey indicates that most ophthalmologists in Japan think OCT findings including CMTs as one of the most important indicators for initial therapeutic intervention [24]. Practically, Snellen chart-based BCVAs are used in Japan. The accuracy and the reliability of the Snellen chart are likely to be less than the ETDRS chart-based BCVAs. Therefore, most Japanese ophthalmologists use OCT findings as the most important assessment indicator [24]. Eyes with a CMT < 250 *μ*m, vitreomacular traction, epiretinal membrane, uveitis, glaucoma, and other retinal diseases, with brain or heart ischemia, were excluded [19, 20, 24]. In addition, patients who did not agree to the high cost of frequent IVA injections were excluded [19, 24].

The injection protocols were the 1+PRN regimen or the 3+PRN regimen. From January 2015 to January 2016, all participants underwent IVA with the 3+PRN regimen because of having followed the main protocols of age-related macular degeneration in our hospital. From January 2016 to January 2017, to save the medical cost, we had revised the regimen from the 3+PRN to the 1+PRN for all patients with DME. From January 2016 to December 2016, either the 1+PRN or the 3+PRN regimen was allocated by each doctor. Thus, both regimens were mixed for the last one year. If the CMT was >300 *μ*m and patients agreed with the possible frequent IVA injections, additional injections were given. However, even if the CMT was <300 *μ*m, when the BCVA deteriorated because of the persistent ME and the patients agreed with the frequent injections, additional injections were given.

If the patients did not agree with the injection regimen, other therapies including vitrectomy and sub-Tenon triamcinolone acetonide (STTA) injection were given. These patients were not included in this study. Sustained release steroid agents were not used because these agents have not been approved in Japan. Thus, STTA injection was selected for treatment of DME instead of sustained release steroid agent injection. The follow-up period was 12 months after the initial IVA treatment. No sight threatening adverse events were observed after the IVA injections.

The BCVAs and the CMTs were measured at the baseline and at 3, 6, and 12 months after the initial IVA injections. The decimal BCVAs were converted to logarithm of the minimum angle of resolution (logMAR) units. A poor baseline BCVA was defined as a BCVA > 0.301 logMAR units, and a good baseline BCVA was defined as a BCVA ≤ 0.301 logMAR units. The center of the macular area was determined to be the center of a 1 mm diameter circle in the ETDRS grid. Scan analysis was performed with the version 5.3 software throughout the study. In case of segmentation errors, the CMTs were measured manually. The examiners measured the macular thickness at the fixation point and at 0.5 mm from the fixation point twice, and the average was used for the data analysis [21]. The DME was classified as serous retinal detachment (SRD), sponge-like DME, or cystoid macular edema (CME) by the OCT images [25]. For the classification of DME, some patients had more than 2 types of DME. In these cases, three masked examiners evaluated the most predominant type and the final characteristic of DME was decided by majority [25].

The clinical data and demographics of the patients before the IVA are shown in Table 1. Fifty-seven eyes had received IVA by the 1+PRN regimen, and 38 eyes had received IVA by the 3+PRN regimen. The age, sex, HbA1c, BCVA, and CMTs were not significantly different between the two groups. In addition, the numbers of patients with the different DME types, i.e., SRD, CME, and sponge-like DME, are presented in Table 2.

All of the procedures conformed to the tenets of the World Medical Association Declaration of Helsinki. A written informed consent form was obtained from all patients. An approval for the study was obtained from the Institutional Review Board of Chiba University Graduate School of Medicine in Japan (number 3541).

### 2.1. Statistical Analyses

The data were expressed as the means ± standard deviations (SDs). The data were analyzed for normality but not randomized because of the retrospective nature. The significance of differences in the data was determined by the Mann-Whitney *U* test, Fisher's exact test, Wilcoxon rank-sum test, or chi-square tests. A *P* value < 0.05 was considered significant.

## 3. Results

### 3.1. Total Number of Injections

**Figure 1:**
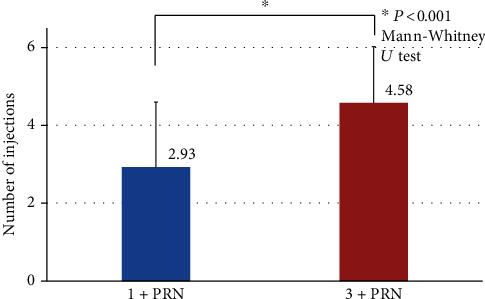
Comparisons of the total number of injections between the 1+PRN and 3+PRN groups at 12 months. The mean total number of injections in the 3+PRN group was 4.6 ± 1.4, which was significantly more than 2.9 ± 1.7 in the 1+PRN group (*P* < 0.001, Mann-Whitney *U* test). PRN: *pro re nata*.

The mean of the total number of IVA injections in the 1+PRN group was 2.93 ± 1.7, which was significantly fewer than 4.6 ± 1.4 in the 3+PRN group (*P* < 0.001; Mann-Whitney *U* test; Figure 1).

### 3.2. Changes in Mean Central Macula Thickness (CMT) from Baseline

**Figure 2:**
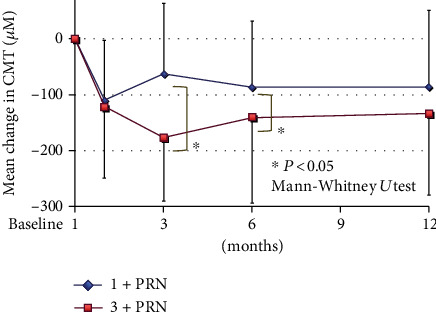
Changes in the mean CMT from baseline to 12 months after the IVA. In the 3+PRN group, the mean CMT was significantly reduced at 3 and 6 months after the IVA compared to that in the 1+PRN group (*P* < 0.001 and *P* = 0.013, respectively; Mann-Whitney *U* test). However, there was no significant difference in the mean CMT between the two groups at 12 months after the IVA. PRN: *pro re nata*; CMT: central macular thickness.

In both the 1+PRN and the 3+PRN groups, the mean CMT was significantly reduced from the baseline at 1, 3, 6, and 12 months after the IVA injections (*P* < 0.001 for all; Wilcoxon rank-sum test; Figure 2). In the 3+PRN group, the mean CMT was significantly reduced at 3 and 6 months after the IVA compared to that in the 1+PRN group (−176.6 ± 113.4 *μ*m vs. −62.6 ± 126.3 *μ*m (*P* < 0.001) and -141.3 ± 152.4 *μ*m vs. -86.1 vs. -117.8 *μ*m (*P* = 0.013), respectively; Mann-Whitney *U* test; Figure 2). However, the difference in the mean CMT between the 3+PRN and 1+PRN groups at 12 months after the IVA was not significant (−133.8 ± 146.2 *μ*m vs. −86.1 ± −136.6 *μ*m; *P* = 0.0969; Mann-Whitney *U* test; Figure 2).

### 3.3. Changes in Mean BCVA from Baseline

**Figure 3:**
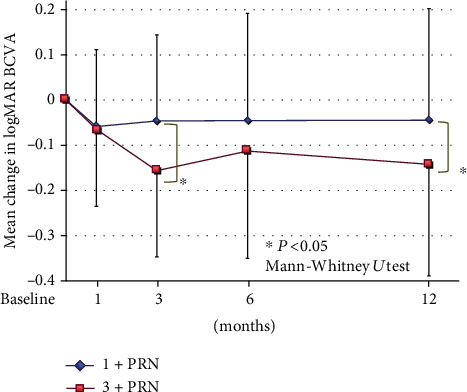
Changes in the mean BCVA (logMAR units) from the baseline to 12 months after the IVA. In the 3+PRN group, the mean BCVA was significantly improved at 3 and 12 months after the IVA compared to that in the 1+PRN group (*P* = 0.0389 and 0.0183, respectively; Mann Whitney *U* test). BCVA: best-corrected visual acuity; PRN: *pro re nata*; logMAR: logarithm of the minimum angle of resolution.

In the 1+PRN group, the BCVAs were significantly better only at 1 month after the IVA injections than at the baseline (*P* = 0.0199; Wilcoxon rank-sum test). In the 3+PRN group, the BCVAs were significantly improved at 1, 3, 6, and 12 months in comparison to the baseline after the IVA injections (*P* = 0.003, *P* < 0.001, *P* = 0.0058, and *P* < 0.001; Wilcoxon rank-sum test; Figure 3).

We also compared mean changes in the BCVA from the baseline after the IVA injections between the two groups. In the 3+PRN group, the mean BCVA was significantly improved at 3 and 12 months after the IVA compared to that in the 1+PRN group (−0.16 ± 0.28 logMAR units vs. -0.05 vs. 0.19 logMAR units (*P* = 0.0389) and −0.14 ± 0.17 logMAR units vs. −0.04 ± 0.25 logMAR units (*P* = 0.0183), respectively; Mann-Whitney *U* test; Figure 3).

### 3.4. Changes in Mean BCVA from Baseline in Eyes Whose Mean Total Number of Injections Was <4

**Figure 4:**
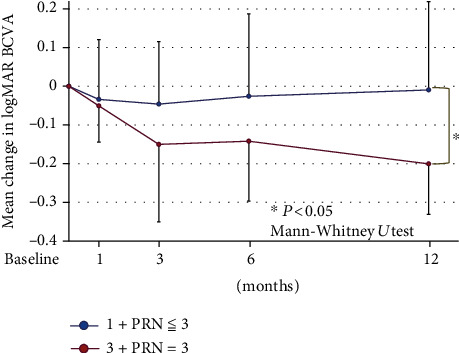
Changes in the mean BCVA from the baseline to 12 months after the IVA injections in eyes in which the mean total number of injections was <4 times. In the 3+PRN group, the mean BCVA was significantly improved at 12 months after the IVA compared to that in the 1+PRN group (*P* = 0.0013, Mann-Whitney *U* test). BCVA: best-corrected visual acuity; PRN: *pro re nata*; logMAR: logarithm of the minimum angle of resolution.

To focus on the DME eyes with a fewer numbers of IVA injections, we compared mean changes in the BCVA from the baseline to 12 months in which the mean total number of IVA injections was <4. In the 1+PRN group, the BCVAs were not significantly improved at any time after the IVA injections. In the 3+PRN group, the BCVAs were significantly improved at 6 and 12 months after the IVA (*P* = 0.0357 and 0.008, respectively; Wilcoxon rank-sum test; Figure 4).

In addition, the total number of injections in the 3+PRN group with ≤3 IVA injections, and the mean BCVA was significantly better at 12 months after the IVA compared to that in the 1+PRN group with the total number of injections ≤ 3 (−0.20 ± 0.13 logMAR units vs. −0.009 ± 0.23 logMAR units; *P* = 0.0013; Mann-Whitney *U* test; Figure 4). Thus, even in the DME eyes in which the mean total number of injections was fewer than 4, the 3+PRN group had significantly better BCVA than the 1+PRN group at 12 months after the IVA injections (Figure 4).

### 3.5. Comparisons of Changes in BCVA from Baseline in Eyes with Poor and Good Baseline BCVA

**Figure 5:**
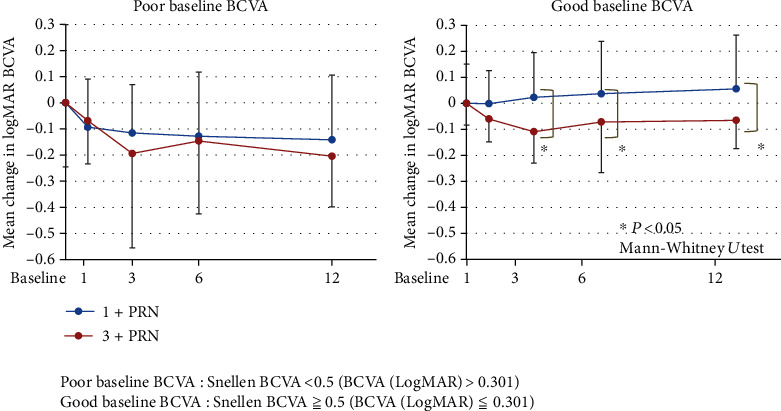
Comparisons of changes in BCVA (logMAR units) from the baseline in the poor baseline BCVA and the good baseline BCVA groups. In eyes with poor baseline BCVA, there was no significant difference in the mean BCVA between the two groups at 12 months after the IVA. However, in eyes with the good baseline BCVA, the mean BCVAs in the 3+PRN group at 3, 6, and 12 months after the IVA were significantly improved compared to those in the 1+PRN group (*P* = 0.0113, *P* = 0.0164, and *P* = 0.044, respectively; Mann-Whitney *U* test). BCVA: best-corrected visual acuity; logMAR: logarithm of the minimum angle of resolution: PRN: *pro re nata*.

In eyes with the poor baseline BCVA, the BCVAs in the 1+PRN group were significantly improved at 1, 3, 6, and 12 months after the IVA in comparison to the baseline BCVA (*P* = 0.0258, 0.0051, 0.0102, and 0.0050, respectively; Wilcoxon rank-sum test; Figure 5). Similarly, the BCVAs in the 3+PRN group were significantly improved at 3, 6, and 12 months after the IVA (*P* = 0.0214, 0.0329, and <0.001, respectively; Wilcoxon rank-sum test) (Figure 5).

In eyes with the good baseline BCVA, the BCVAs in the 1+PRN group were not significantly improved from the baseline BCVA at any time (Figure 5). However, the BCVAs in the 3+PRN group were significantly improved at 1, 3, 6, and 12 months from the baseline BCVA (*P* = 0.0367, 0.0071, 0.0231, and 0.034, respectively; Wilcoxon rank-sum test; Figure 5).

Furthermore, in eyes with the poor baseline BCVA, there was no significant difference in the mean BCVA between the two groups at 12 months after the IVA (Figure 5). However, in eyes with the good baseline BCVA, the mean BCVAs in the 3+PRN group at 3, 6, and 12 months after the IVA were significantly better than those in the 1+PRN group (*P* = 0.0113, 0.0164, and 0.044, respectively; Mann-Whitney *U* test) (Figure 5).

### 3.6. Comparison of Total Number of Injections in Eyes with CME and Sponge-Like DME at 12 Months

**Figure 6:**
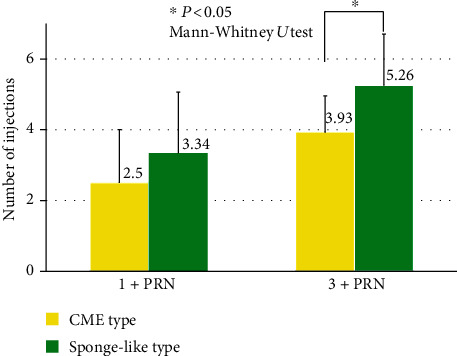
Comparisons of the total number of injections in eyes with CME to that with sponge-like DME at 12 months. In the 1+PRN group, there was no significant difference between CME and sponge-like DME (2.5 ± 1.5 vs. 3.3 ± 1.7; *P* = 0.075, Mann-Whitney *U* test). However, in the 3+PRN group, the total number of injections in the eyes with sponge-like DME was significantly more than that in eyes with CME (5.3 ± 1.4 vs. 3.9 ± 1.0; *P* = 0.0063, Mann-Whitney *U* test). PRN: *pro re nata*; CME: cystoid macular edema.

To examine the effect of IVA on different types of DME, we classified DME as sponge-like ME or CME. Because of the small number of the SRD type, the SRD type was not included in this analysis. The number of patients in each group was not significantly different between the two DME types (*P* = 0.7877; chi-square test; Table 2). In the 1+PRN group, there was no significant difference of the total number of injections between CME and sponge-like DME (2.5 ± 1.5 vs. 3.3 ± 1.7; *P* = 0.075, Mann-Whitney *U* test) (Figure 6). However, the total number of injections of the sponge-like DME in the 3+PRN group was significantly more than that in the CME group (5.3 ± 1.4 vs. 3.9 ± 1.0; *P* = 0.0063, Mann-Whitney *U* test; Figure 6).

### 3.7. Changes in Mean BCVA and Mean CMT from Baseline to 12 Months after IVA for CME and Sponge-Like DME

**Figure 7:**
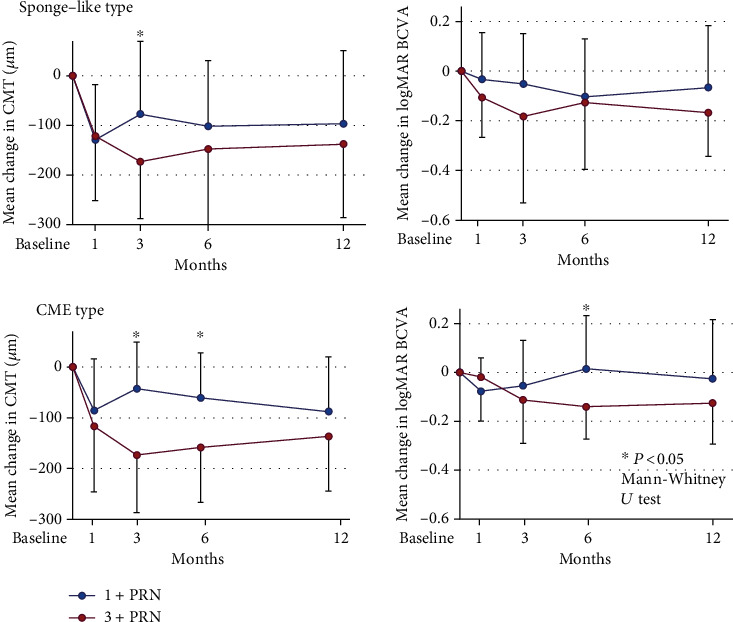
Changes in the mean BCVA (logMAR units) and the mean CMT from baseline to 12 months after the IVA for CME and sponge-like DME. There were no significant differences both in the mean BCVA and in the mean CMT between the 1+PRN and 3+RPN in eyes with CME and sponge-like DME at 12 months. PRN: *pro re nata*; CMT: central macular thickness; BCVA: best-corrected visual acuity; logMAR: logarithm of the minimum angle of resolution.

In eyes with sponge-like DME, the BCVA in the 1+PRN group was significantly improved only at 6 months after the IVA (*P* = 0.0096; Wilcoxon rank-sum test; Figure 7). However, the BCVA in the 3+PRN group was significantly improved at 1, 3, and 12 months after the IVA (*P* = 0.0144, *P* = 0.0310, and *P* = 0.0016, respectively; Wilcoxon rank-sum test; Figure 7). The CMTs in the 1+PRN group were significantly reduced at 1, 3, 6, and 12 months after the IVA (*P* < 0.001, *P* = 0.0031, *P* < 0.001, and *P* = 0.0021, respectively; Wilcoxon rank-sum test; Figure 7). The CMTs in the 3+PRN group were also significantly reduced at 1, 3, 6, and 12 months after the IVA (*P* = 0.0049, *P* < 0.001, *P* = 0.0048, and *P* = 0.00025, respectively; Wilcoxon rank-sum test; Figure 7).

In eyes with CME, the BCVA in the 1+PRN group was significantly improved only at 1 month after the IVA (*P* = 0.0218; Wilcoxon rank-sum test; Figure 7). The BCVA in the 3+PRN group was significantly improved at 3, 6, and 12 months after the IVA (*P* = 0.0413, *P* = 0.0039, and *P* = 0.0231, respectively; Wilcoxon rank-sum test; Figure 7).

The CMT in the 1+PRN group was significantly reduced at 1, 6, and 12 months after the IVA (*P* = 0.0025, *P* = 0.0051, and *P* = 0.0019, respectively; Wilcoxon rank-sum test; Figure 7). The CMT in the 3+PRN group was significantly reduced at 1, 3, 6, and 12 months after the IVA (*P* = 0.0038, *P* < 0.001, *P* < 0.001, and *P* < 0.001, respectively; Wilcoxon rank-sum test; Figure 7).

There were no significant differences in the mean BCVA and in the mean CMT between the 1+PRN and 3+RPN in eyes with CME and sponge-like DME at 12 months (Figure 7).

## 4. Discussion

The results indicated that IVA injections followed by the 3+PRN regimen significantly improved the BCVAs and the CMT more than those followed by the 1+PRN protocol in eyes with DME. Thus, the IVA injections using the 1+PRN protocol were not effective in treating patients with DME in the everyday clinical practice.

These results are not consistent with the results of earlier studies on the effectiveness of anti-VEGF injections for ME associated with retinal vein occlusion [21, 26–29]. These earlier results suggested that the effects of the 1+PRN anti-VEGF regimen were not significantly different from those of the 3+PRN regimen in improving the BCVAs and CMTs at 6 or 12 months in eyes with ME associated with retinal vein occlusion [21, 26–29]. In addition, the total number of injections with the 1+PRN protocols was significantly fewer than that with the 3+PRN protocol [21, 26–29]. Thus, all of these studies concluded that the 1+PRN anti-VEGF injection regimen was recommended in eyes with ME associated with retinal vein occlusion [21, 26–29]. On the other hand, in eyes with DME of this study, the 1+PRN IVA injection protocol did not significantly improve the BCVAs at 12 months after the IVA. Thus, the 3+PRN IVA injection is better even though the total number of IVA injections was significantly higher than that of the 1+PRN IVA injection regimen for the treatment of DME.

The fewer number of injections (less than 4) indicated that only the initial three monthly consecutive injections led to a significant improvement of the BCVAs (11/38 eyes; 29%) compared to the 1+PRN fewer than 4 injections (35/57 eyes; 61%). Gonzalez et al. suggest that the BCVA at three months after the IVR+prompt/deferred laser photocoagulation was significantly correlated with the BCVAs at 5 years after the initial treatment [30]. Taken together with our findings, the initial three injections may be recommended to maintain better BCVAs for a longer period.

Baker et al. reported that in eyes with good baseline BCVA, there was no significant improvement of the BCVAs at 24 months after IVA injections with the PRN regimen [31]. However, the results of eyes with good baseline BCVAs in our cohort indicated that the 3+PRN regimen significantly improved BCVAs at 12 months after the IVA, but not in the 1+PRN group at any time. Thus, even with good baseline BCVAs, the 3+PRN IVA injection regimen can improve the BCVAs in eyes with DME.

We also examined the effects of IVA on different types of DME. Because the number of patients was small, the data of the SRD type could not be analyzed, but the results of several recent studies are consistent; i.e., anti-VEGF injections were significantly more effective for the improvement of the SRD types of DME [19, 20, 32, 33]. We did compare the effects of IVA on eyes with the sponge-like DME and the CME type. Although more injections were required for the sponge-like DME than for the CME type, the 3+PRN IVA injection protocol significantly improved the BCVAs at 12 months after the IVA in both sponge-like and CME types, but the 1+PRN protocol did not improve the BCVAs at 12 months in both types. Taken together, the 3+PRN IVA injection regimen is effective for both sponge-like and CME types of DME.

In Japan, most ophthalmologists select anti-VEGF treatment as the first-line therapy for DME, but the preferable protocol is the 1+PRN regimen [25]. In this Japanese survey, however, if both the BCVAs and the CMTs deteriorated again after the loading treatment, retreatment was performed especially with the 1+PRN protocol. Although it is not the best protocol, the results of the study indicate that the 3+PRN regimen is more effective than the 1+PRN regimen for the IVA treatment of DME. Because financial limitation is the most important difficulty for the 3+PRN regimen, combination therapies with IVA such as STTA may be required for reducing the total number of injections. Sustained release steroid agents, which are frequently used in Europe [5–9], are not used in Japan because these agents have not been approved in Japan yet. Instead of these agents, intravitreal and/or sub-Tenon triamcinolone acetonide injections are frequently used in Japan [25].

In this study, the OCT findings and the CMTs are used as the most important assessment indicators for IVA injections [25]. Thus, the OCT device used in this study, SD-OCT, is an important factor for the interpretation of the results of this study. If the different OCT devices including 3D-OCT and swept source OCT are used in this study, the results may be changed. Thus, the results of this study should be interpreted with caution because consideration of IVA injections was based on the OCT findings.

There are other limitations in this study. First, it was a retrospective study and selection bias cannot be removed completely. Second, the retreatment criteria were not fixed beforehand. Third, the efficacies of IVA treatment in both the BCVAs and the CMTs deteriorated again after the loading treatment, regardless of the protocols used (1+PRN or 3+PRN) because of smaller total number of injections compared to that in the clinical trials. Thus, we cannot determine that the 3+PRN regimen is the best treatment regimen. Further prospective randomized clinical studies are required to confirm the best treatment protocol for patients with DME.

In conclusion, the 3+PRN IVA injection regimen can significantly improve the BCVAs and the CMTs at 12 months after the IVA compared to the 1+PRN protocol in eyes with DME. The effectiveness of the 3+PRN protocol is not dependent on the type of DME and baseline BCVA. Thus, although the total number of injections is more than that in the 1+PRN protocol, we recommend the 3+PRN IVA injection protocol for the treatment of DME in a clinical practice.

## Figures and Tables

**Table 1:** Baseline characteristics of the patients.

Baseline	1+PRN group	3+PRN group	*P*
Number (eyes)	57	38	NA
Age (years)	61.7 ± 12.1	65.1 ± 9.2	*P* = 0.32
Gender (men/women)	34/23	24/14	*P* = 0.83
HbA1c (%)	7.67 ± 2.24	7.55 ± 1.04	*P* = 0.065
BCVA (logMAR)	0.42 ± 0.34	0.45 ± 0.29	*P* = 0.73
CMT (*μ*m)	500.5 ± 120.3	537.7 ± 92.7	*P* = 0.067

BCVA: best-corrected visual acuity; CMT: central macular thickness; logMAR: logarithm of the minimum angle of resolution.

**Table 2:** Different types of DME on OCT.

	1+PRN (eyes)	3+PRN (eyes)
Sponge-like type	32	19
CME type	20	16
SRD type	5	3

OCT: optical coherence tomography; CME: cystoid macular edema; SRD: serous retinal detachment; PRN: *pro re nata*.

## Data Availability

The data used to support the findings of this study is available upon request from the corresponding author.

## References

[1] O'Doherty M., Dooley I., Hickey-Dwyer M. (2008). Interventions for diabetic macular oedema: a systematic review of the literature. *British Journal of Ophthalmology*.

[2] Yau J. W., Rogers S. L., Kawasaki R. (2012). Global prevalence and major risk factors of diabetic retinopathy. *Diabetes Care*.

[3] Zur D., Iglicki M., Loewenstein A. (2019). The role of steroids in the management of diabetic macular edema. *Ophthalmic Research*.

[4] Iglicki M., Lavaque A., Ozimek M. (2018). Biomarkers and predictors for functional and anatomic outcomes for small gauge pars plana vitrectomy and peeling of the internal limiting membrane in naïve diabetic macular edema: the VITAL study. *PLoS One*.

[5] Iglicki M., Busch C., Zur D. (2019). Dexamethasone implant for diabetic macular edema in naive compared with refractory eyes: the international retina group real-life 24-month multicenter study. The IRGREL-DEX study. *Retina*.

[6] Iglicki M., Zur D., Busch C., Okada M., Loewenstein A. (2018). Progression of diabetic retinopathy severity after treatment with dexamethasone implant: a 24-month cohort study the 'DR-Pro-DEX Study'. *Acta Diabetologica*.

[7] for the International Retina Group (IRG), Iglicki M., Zur D. (2019). TRActional DIabetic reTInal detachment surgery with co-adjuvant intravitreal dexamethasONe implant: the TRADITION STUDY. *Acta Diabetologica*.

[8] Zur D., Iglicki M., Sala-Puigdollers A. (2020). Disorganization of retinal inner layers as a biomarker in patients with diabetic macular oedema treated with dexamethasone implant. *Acta Ophthalmologica*.

[9] Mello Filho P., Andrade G., Maia A. (2018). Effectiveness and safety of intravitreal dexamethasone implant (Ozurdex) in patients with diabetic macular edema: a real-world experience. *Ophthalmologica*.

[10] Sultan M. B., Zhou D., Loftus J., Dombi T., Ice K. S., Macugen 1013 Study Group (2011). A phase 2/3, multicenter, randomized, double-masked, 2-year trial of pegaptanib sodium for the treatment of diabetic macular edema. *Ophthalmology*.

[11] Mitchell P., Bandello F., Schmidt-Erfurth U. (2011). The RESTORE study: ranibizumab monotherapy or combined with laser versus laser monotherapy for diabetic macular edema. *Ophthalmology*.

[12] Diabetic Retinopathy Clinical Research Network, Elman M. J., Qin H. (2012). Intravitreal ranibizumab for diabetic macular edema with prompt versus deferred laser treatment: three-year randomized trial results. *Ophthalmology*.

[13] Ishibashi T., Li X., Koh A. (2015). The REVEAL study: ranibizumab monotherapy or combined with laser versus laser monotherapy in Asian patients with diabetic macular edema. *Ophthalmology*.

[14] Do D. V., Nguyen Q. D., Boyer D. (2012). One-year outcomes of the da Vinci Study of VEGF Trap-Eye in eyes with diabetic macular edema. *Ophthalmology*.

[15] Brown D. M., Schmidt-Erfurth U., Do D. V. (2015). Intravitreal Aflibercept for diabetic macular edema: 100-week results from the VISTA and VIVID studies. *Ophthalmology*.

[16] Schmidt-Erfurth U., Lang G. E., Holz F. G. (2014). Three-year outcomes of individualized ranibizumab treatment in patients with diabetic macular edema: the RESTORE extension study. *Ophthalmology*.

[17] Elman M. J., Ayala A., Bressler N. M. (2015). Intravitreal ranibizumab for diabetic macular edema with prompt versus deferred laser treatment: 5-year randomized trial results. *Ophthalmology*.

[18] Ross E. L., Hutton D. W., Stein J. D. (2016). Cost-effectiveness of aflibercept, bevacizumab, and ranibizumab for diabetic macular edema treatment: analysis from the diabetic retinopathy clinical research network comparative effectiveness trial. *JAMA Ophthalmology*.

[19] Kaiho T., Oshitari T., Tatsumi T. (2017). Efficacy of one-year treatment with aflibercept for diabetic macular edema with practical protocol. *BioMed Research International*.

[20] Shimizu N., Oshitari T., Tatsumi T. (2017). Comparisons of Efficacy of Intravitreal Aflibercept and Ranibizumab in Eyes with Diabetic Macular Edema. *BioMed Research International*.

[21] Nagino Y., Tatsumi T., Oshitari T. (2020). Comparison of one injection to three monthly injections of anti-vascular endothelial growth factor agents for macular oedema associated with branch retinal vein occlusion. *HSOA Journal of Ophthalmology & Clinical Research*.

[22] Ebneter A., Waldmeier D., Zysset-Burri D. C., Wolf S., Zinkernagel M. S. (2017). Comparison of two individualized treatment regimens with ranibizumab for diabetic macular edema. *Graefe's Archive for Clinical and Experimental Ophthalmology*.

[23] James D. G. P., Mitkute D., Porter G., Vayalambrone D. (2019). Visual outcomes following intravitreal ranibizumab for diabetic macular edema in a pro re nata protocol from baseline: a real-world experience. *Asia-Pacific Journal of Ophthalmology*.

[24] Sugimoto M., Tsukitome H., Okamoto F. (2019). Clinical preferences and trends of anti-vascular endothelial growth factor treatments for diabetic macular edema in Japan. *Journal of Diabetes Investigation*.

[25] Otani T., Kishi S., Maruyama Y. (1999). Patterns of diabetic macular edema with optical coherence tomography. *American Journal of Ophthalmology*.

[26] Ito Y., Saishin Y., Sawada O. (2015). Comparison of single injection and three monthly injections of intravitreal bevacizumab for macular edema associated with branch retinal vein occlusion. *Clinical Ophthalmology*.

[27] Ahn S. J., Ahn J., Woo S. J., Park K. H. (2013). Initial dose of three monthly intravitreal injections versus PRN intravitreal injections of bevacizumab for macular edema secondary to branch retinal vein occlusion. *BioMed Research International*.

[28] Miwa Y., Muraoka Y., Osaka R. (2017). Ranibizumab for macular edema after branch retinal vein occlusion: one initial injection versus three monthly injections. *Retina*.

[29] Bayat A. H., Çakır A., Özturan S. G., Bölükbaşı S., Erden B., Elçioğlu M. N. (2018). Comparison of one and three initial monthly intravitreal ranibizumab injection in patients with macular edema secondary to branch retinal vein occlusion. *International Journal of Ophthalmology*.

[30] Gonzalez V. H., Campbell J., Holekamp N. M. (2016). Early and long-term responses to anti-vascular endothelial growth factor therapy in diabetic macular edema: analysis of protocol I data. *American Journal of Ophthalmology*.

[31] Baker C. W., Glassman A. R., Beaulieu W. T. (2019). Effect of initial management with aflibercept vs laser photocoagulation vs observation on vision loss among patients with diabetic macular edema involving the Center of the Macula and Good Visual Acuity: a randomized clinical trial. *JAMA*.

[32] Sophie R., Lu N., Campochiaro P. A. (2015). Predictors of functional and anatomic outcomes in patients with diabetic macular edema treated with ranibizumab. *Ophthalmology*.

[33] Korobelnik J. F., Lu C., Katz T. A. (2019). Effect of baseline subretinal fluid on treatment outcomes in VIVID-DME and VISTA-DME studies. *Ophthalmology Retina*.

